# Myocardial changes in incident haemodialysis patients over 6-months: an observational cardiac magnetic resonance imaging study

**DOI:** 10.1038/s41598-017-14481-y

**Published:** 2017-10-25

**Authors:** Elaine Rutherford, Kenneth Mangion, Christie McComb, Elizabeth Bell, Samantha Cockburn, Mohammed Talle, Giles Roditi, Paul Welsh, Rosemary Woodward, Aleksandra Radjenovic, Allan D. Struthers, Alan G. Jardine, Colin Berry, Rajan K. Patel, Patrick B. Mark

**Affiliations:** 1 Institute of Cardiovascular and Medical Sciences, University of Glasgow, BHF Building, 126 University Place, Glasgow, G12 8TA UK; 2 Glasgow Renal & Transplant Unit, Queen Elizabeth University Hospital, NHS Greater Glasgow & Clyde, Glasgow, UK; 3 Department of Radiology, Glasgow Royal Infirmary, NHS Greater Glasgow & Clyde, Glasgow, UK; 4 School of Medicine, University of Dundee, Ninewells Hospital & Medical School, Dundee, UK

## Abstract

Patients commencing on haemodialysis (HD) have an increased risk of cardiovascular events in the first year after starting HD compared to those patients established on HD longer. Left ventricular (LV) hypertrophy and abnormal myocardial strain predict mortality. There may be changes in the myocardium of incident HD patients over a 6-month period of HD which may explain changes in cardiovascular risk. We used CMR to consider changes in LV mass, myocardial strain and T1 mapping. We examined changes in pre-dialysis highly sensitive troponin T. 33 patients undergoing HD for <12 months were recruited. Participants underwent CMR at baseline and after 6-months of standard care. 6-months of HD was associated with reduction in LV mass index (Baseline: 78.8 g/m^2^ follow up: 69.9 g/m^2^, p = <0.001). LV global longitudinal strain also improved (Baseline: −17.9%, follow up: −21.6%, p = <0.001). Change in T1 time was not significant (Baseline septal T1 1277.4 ms, follow up 1271.5 p = 0.504). Highly sensitive troponin T was lower at follow up (Baseline 38.8 pg/L, follow up 30.8 pg/L p = 0.02). In incident HD patients, 6-months of HD was associated with improvements in LV mass, strain and troponin. These findings may reflect improvement in known cardiac tissue abnormalities found in patients over the first year of HD.

## Introduction

Cardiovascular disease is a major cause of morbidity and mortality amongst hemodialysis (HD) patients. Echocardiography studies demonstrate that up to three quarters of patients commencing HD have left ventricular hypertrophy (LVH)^1^. Presence of LVH on HD commencement is associated with increased risk of cardiovascular and all-cause mortality^2,3^. The pathophysiology of myocardial disease in HD is complex and in addition to myocardial hypertrophy, involves pump dysfunction, ventricular dilation and myocardial fibrosis^4,5^. This pathophysiology of cardiovascular disease in HD patients is incompletely understood, especially why incident HD patients have greater risk of sudden death over the first year of HD than those established on HD for longer^6^.

Cardiac Magnetic Resonance imaging (CMR) is a leading tool for examining the hearts of HD patients. CMR is highly reproducible in these patients where changing volume status make echocardiography less reliable^7^. CMR is better able to characterize cardiac tissue. Although it is not possible to use gadolinium contrast in these patients because of concerns regarding nephrogenic systemic fibrosis^8^, newer non-contrast techniques to characterize cardiac tissue are emerging. Recently, native T1 mapping identified abnormalities in uremic hearts compared to healthy volunteers, and co-morbid matched patients^9,10^. These abnormalities are in keeping with those demonstrated using gadolinium contrast and have been verified in CKD patients with adequate renal function to permit gadolinium use^11,12^.

Myocardial strain, often measured as global longitudinal strain (GLS), a measure of myocardial deformation throughout the cardiac cycle, is more directly associated with cardiomyocyte metabolism than LV ejection fraction (LVEF)^13^. Reduction in magnitude of peak strain is associated with reduced myocardial contraction and could be associated with fibrosis^14^. CMR GLS has been demonstrated to be impaired in several renal populations, including in CKD, incident and prevalent HD patients, as well as correlating with cardiac histology in animal models of kidney failure^9,10,12,15^.

We undertook an observational study to interrogate the hearts of incident HD patients over a 6-month period. We sought to identify detectable changes in cardiac structure, function and composition using CMR. We assessed associations between these and clinical data. We hypothesized that haemodialysis would affect myocardial contractility (measured by myocardial strain) and could reduce or reverse diffuse fibrosis, measured by native T1 mapping. We hypothesized changes in biomarkers associated with myocardial stress would occur (highly-sensitive troponin T (hs-TropT) and N-Terminal Pro-B type Natriuretic Peptide (NT-ProBNP)).

## Results

### Participants

Thirty-four patients consented to participate; 32 participants completed baseline imaging, one participant died after consenting but prior to study visit, another withdrew consent before imaging. Twenty-four participants (75% of consented participants) completed baseline and follow-up visits. Six participants were transplanted during the study and 2 participants withdrew consent. No participants died during follow up. Mean participant age was 61.2 ± 13.4 years, 62.5% were male. Median duration of HD at baseline was 5 months (interquartile range (IQR) 3.8–8 months). Baseline demographic data for study participants completing the study are shown in Table 1.

**Table 1:** Baseline Participant Demographic Data. Data is shown as mean ± standard deviation, % [number of participants] or median (interquartile range).

Patient Characteristics	Patients [N = 24]
**Age (years)**	61.2 ± 13.4
**Male (%)**	62.5 [15]
**BMI (kg/m** ^**2**^ **)**	24 (21.7–29.5)
**Duration RRT (months)**	5 (3.8–8)
**Primary Renal Diagnosis**	
Diabetic Nephropathy (%)	29.2 [7]
Renovascular Disease (%)	16.7 [4]
Glomerulonephritis (%)	12.5 [3]
Polycystic Kidney Disease (%)	4.2 [1]
Other (%)	20.7 [5]
Unknown (%)	16.7 [4]
**Past Medical History**	
Diabetes (%)	29.2 [7]
Hypertension (%)	54.2 [13]
Stroke (%)	16.7 [4]
Myocardial Infarction (%)	12.5 [3]
Ischaemic Heart Disease (%)	20.7 [5]
**Drug history**	
B-Blocker	3 [13]
ACE-Inhibitor	19 [79]

### Left Ventricular Mass and Function

**Figure 1:**
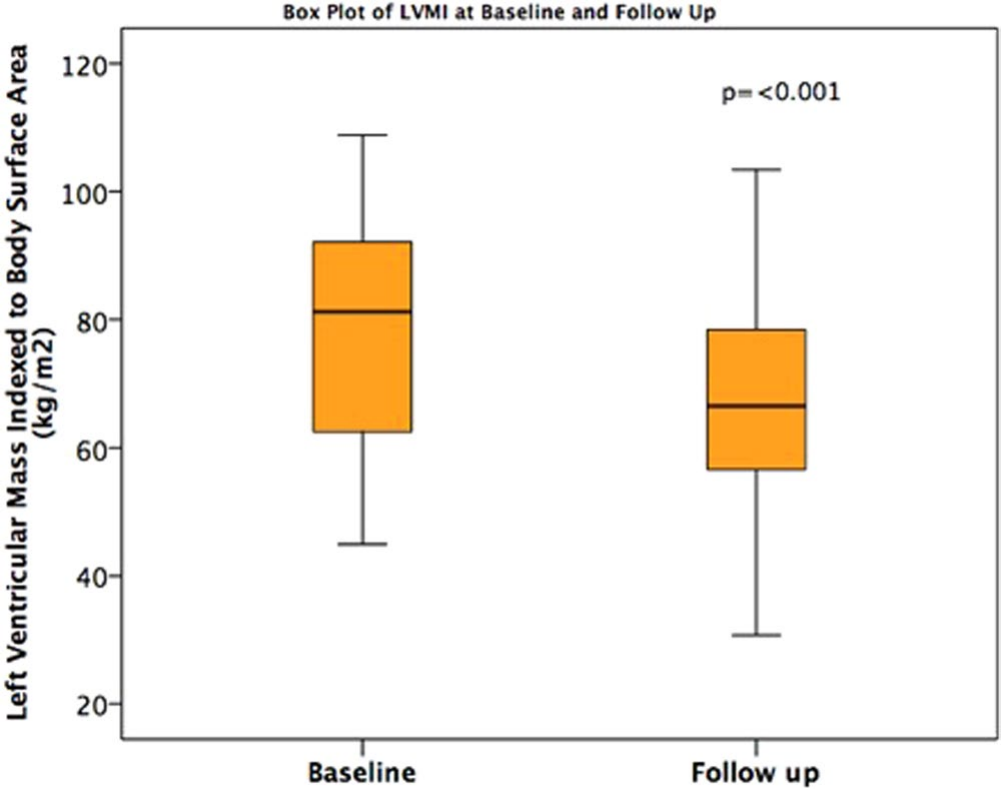
Box plot of Left Ventricular Mass Index at baseline and follow up.

LV mass indexed to body surface area (LVMI) reduced following 6 months of HD. Baseline LVMI was 78.8 ± 18.6 g/m^2^ versus follow up LVMI 69.9 ± 19.3 g/m^2^, p = <0.001 (Fig. 1). There was no change in LVEF between baseline and follow up visits (baseline LVEF 64.3 ± 10.0%, follow up LVEF 61.7 ± 9.9 p = 0.13). End diastolic volumes (EDV) and end systolic volumes (ESV) were unchanged from baseline to follow up. Table 2 summarises clinical, CMR and biomarker indices at baseline and follow up (Table 2).

**Table 2:** Clinical, biochemical and CMR parameters at baseline and follow up.

Variable	Baseline [N = 24]	Follow Up [N = 24]	P value
Systolic BP (mmHg)	140 ± 19	140 ± 21	0.99
Diastolic BP (mmHg)	78 (66–83)	73 (63–81)	0.29
Haemoglobin (mg/dL)	109 (99–118)	109 (104–115)	0.65
Urea Reduction Ratio (%)	71.8 ± 8.4	72.3 ± 7.3	0.70
30-day mean ultrafiltration volume (ml)	1718 ± 905	1703 ± 937	0.93
Post HD weight (kg)	65.6 (60.5–82.4)	66.2 (59.7–80.4)	0.15
Albumin (g/L)	33.5 (32–35)	35 (33.8–36.3)	0.08
Phosphate (mmol/L)	1.73 ± 5.4	1.69 ± 5.3	0.59
Corrected Calcium (mmol/L)	2.30 ± 0.12	2.29 ± 0.13	0.96
Highly sensitive Troponin T (pg/L)*	**38.8 (24.5**–**49.5)**	**30.8 (18.9**–**57.6)**	**0.02**
NT-ProBNP (pg/ml)*	3348 (1718–5112)	2317 (1559–7028)	0.43
Ejection Fraction (%)	64.3 ± 10.0	61.7 ± 9.9	0.13
End Diastolic Volume (ml)	143.2 (136.7–157.6)	146.9 (127–168)	0.78
End Systolic Volume (ml)	50.9 (40.7–58.4)	53.1 (42.2–64.6)	0.16
Stroke Volume (ml)	95.9 ± 21.9	90.6 ± 17.5	0.15
**Left Ventricular Mass (g)**	**139.1 (112.4**–**163.7)**	**114.0 (100.9**–**155)**	**<0.001**
**LVMI (g/m** ^**2**^ **)**	**78.8** ± **18.6**	**69.9** ± **19.3**	**<0.001**
**Global Longitudinal Strain (%)**	**−17.9** ± **5.3**	**−21.6** ± **6.1**	**<0.001**
**Strain Rate (s** ^**−1**^ **)**	**0.97** ± **0.28**	**1.184** ± **0.46**	**0.01**
Early Diastolic Strain Rate (s^−1^)	1.04 ± 0.33	1.16 ± 0.43	0.15
Global T1 Time (ms)	1263.4 ± 24.8	1259.3 ± 32.2	0.59
Septal T1 Time (ms)	1277.4 ± 28.2	1271.5 ± 35.4	0.50

Data is shown as mean ± standard deviation, or median (interquartile range).

Abbreviations: LVMI; left ventricular mass indexed to body surface area, NT-ProBNP; N-terminal pro beta natriuretic peptide.

^*^NT-ProBNP and hs-tropT were available for 22 participants only.

### Native T1 Times

Native T1 times were unchanged following 6 months of HD. Baseline global T1 time: 1263.4 ± 24.8 ms, follow up global T1 time; 1259.3 ± 32.2 ms, p = 0.59. Mean septal T1 time baseline; 1277.4 ± 28.2 ms, follow up 1271.5 ± 35.4 ms, p = 0.50.

### Myocardial Strain

**Figure 2:**
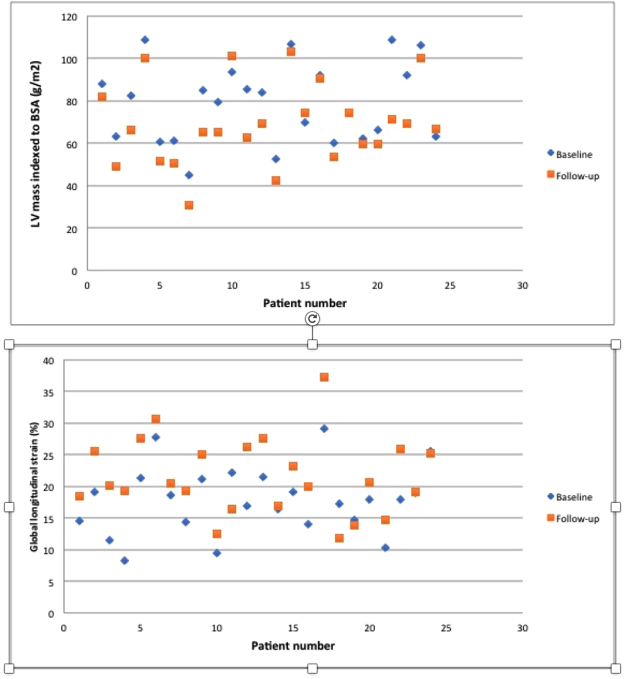
Scatter plots of Left Ventricular Mass Index and Global longitudinal strain at baseline and follow-up.

Reflecting improved myocardial compliance, magnitude of GLS and strain rate both increased following 6 months of HD. Baseline GLS: −17.9 ± 5.3%, follow up GLS-21.6 ± 6.1%, p = <0.001 (Fig. 2). Baseline strain rate: 0.97 ± 0.28 s^−1^, follow up strain rate 1.184 ± 0.46 s^−1^, p = 0.01. There was no significant change in early diastolic strain rate from baseline to follow up. Baseline early diastolic strain rate 1.04 ± 0.33 s^−1^, follow up early diastolic strain rate 1.16 ± 0.43 s^−1^, p = 0.14.

### Biomarkers

There was no change in NT-proBNP from baseline to follow up (median baseline NT-proBNP 3348 (IQR 1718–5112) pg./ml, follow up 2317 (IQR 1559–7028 pg/ml p = 0.44). There was a reduction in hs-tropT from baseline to follow up (Baseline median hs-TropT: 38.8 (IQR 24.5–49.5) pg/L, follow up: 30.8 (IQR 18.9–57.6) pg/L, p = 0.02).

### Clinical Data

There was no change in URR, blood pressure (BP) or ultrafiltration volumes from baseline to 6 months follow up. Weight at end of dialysis the session before imaging was unchanged from baseline to follow up. There were no significant changes in any routinely collected blood test results during the study.

### Relationships between T1 times and left ventricular indices

There were correlations between baseline LVMI and baseline septal T1 time (Pearson’s R = 0.566, p = 0.04). At follow up, this correlation was not statistically significant (Pearson’s R = 0.396, p = 0.055). Similarly, global T1 time at baseline correlated with baseline LVMI (Pearson’s R = 0.585 p = 0.003), however follow up global T1 time was not correlated with follow up LVMI (Pearson’s R = 0.350, p = 0.09). The difference in LVMI over the study period also correlated with difference in septal T1 time (R = 0.454, p = 0.03), but not difference in global T1 time (R = 0.375, p = 0.07). There was no association demonstrated between EDV, ESV or LVEF and T1 times.

### Relationships between myocardial strain and left ventricular indices

At baseline, GLS was related to LVEF (Pearson’s R = −0.601, p = 0.002), LVMI (Pearson’s R = 0.621, p = 0.001), EDV (Spearman’s R = 0.470, p = 0.02) and ESV (Spearman’s R = −0.577, p = 0.03. At follow up similar associations with GLS were demonstrated for LVEF and LVMI but not EDV or ESV: GLS and LVEF (Pearson’s R = −0.425, p = 0.04), LVMI (Pearson’s R = 0.496, p = 0.01), EDV (Spearman’s R = 0.264, p = 0.21). There was no correlation between difference in LVMI and the difference in GLS.

### Relationship between Myocardial Strain and native T1

At baseline, GLS correlated with global native T1 relaxation time (Pearson’s R = 0.461, p = 0.02) and with septal T1 (Pearson’s R = 0.434, p = 0.03). At follow up this relationship was not seen: follow up GLS and global native T1 (Pearson’s R = 0.101, p = 0.64), follow up GLS and septal T1 (Pearson’s R = 0.062, p = 0.77). There was no correlation between change in GLS and change in native T1 times. There were no relationships between strain rate or early diastolic strain rate and native T1 time.

### Relationship between Biomarkers and CMR Findings

At both baseline and follow up, there were no correlations demonstrated between any measure of NT-proBNP, hs-tropT or any LV indices, native T1 or myocardial strain.

### Patients where Septal T1 times reduced compared to where it did not

**Figure 3:**
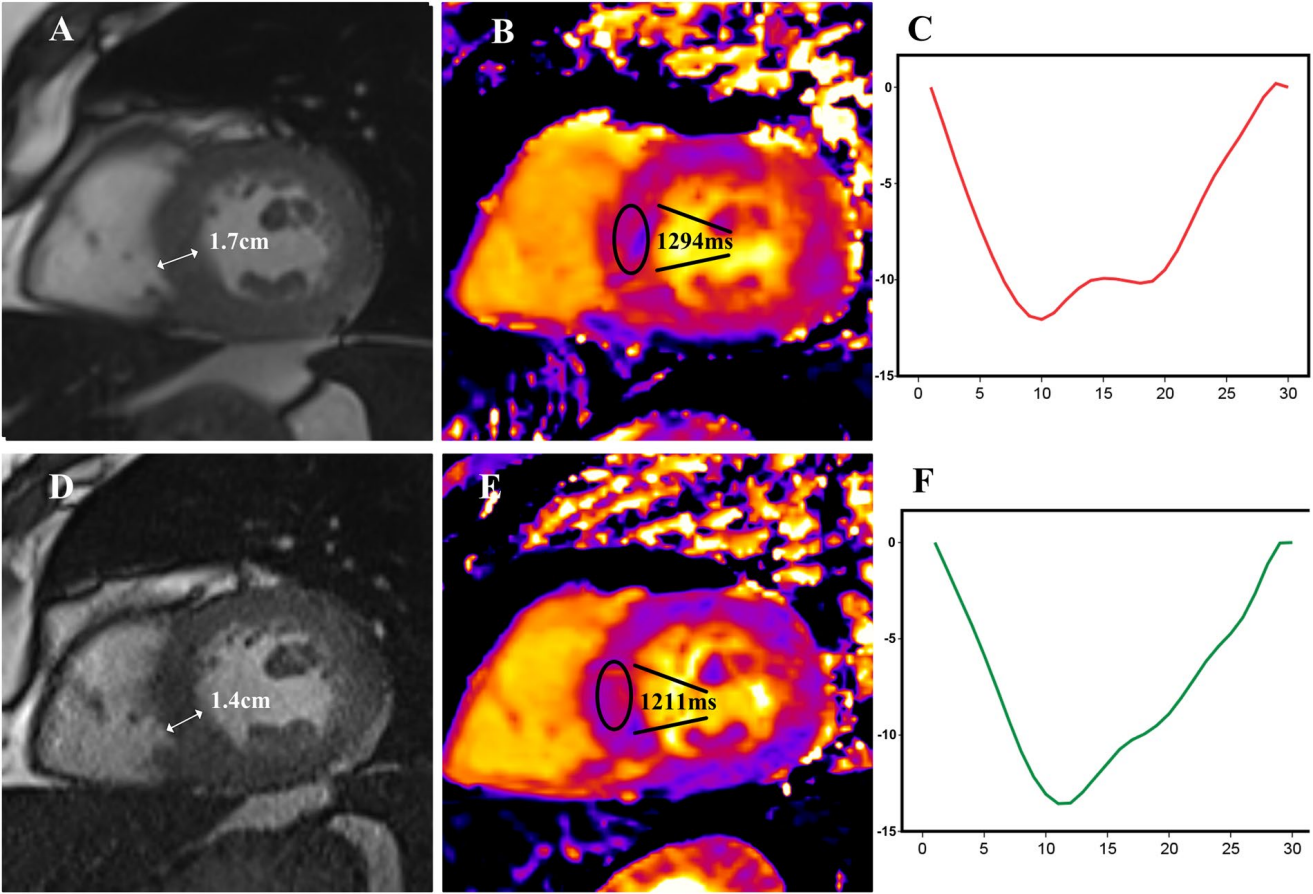
Typical changes in a study participant whose cardiac parameters improved over the duration of the study. Panels A–C are baseline images, panels D–F are follow up images. Panel A shows a short axis view of the left ventricle on baseline imaging, the myocardial thickness measures 1.7 cm, by follow up (panel D) the diameter had reduced to 1.4 cm. Panel B shows a typical T1 map obtained during the study, an interventricular septal segment has been drawn on and shows a T1 time of 1294 ms, by follow up (panel E) the corresponding segment had a noticeable reduction in T1 time to 1211 ms. Panel C shows a representative graph of global longitudinal strain; the horizontal axis is time throughout the cardiac cycle and the vertical axis shows amount of myocardial displacement. On comparison of panel C with panel F the displacement is greater in panel F, representing an improvement in myocardial strain mechanics over the study follow up period.

Overall there was a pattern of improvement in cardiac parameters in some participants but not others. Figure 3 illustrates changes seen in a typical patient whose parameters improved.

Septal T1 times improved in 66.7% of patients. Patients whose septal T1 time improved had higher baseline septal T1 compared with those whose T1 time did not improve (1288.1 ± 28.1 ms, versus 1256.0 ± 11.6 ms, p = 0.006). By follow up, septal T1 time was lower in the ‘improving’ group compared to the ‘non-improving’ group (follow up septal T1 time improvers 1258.8 ± 22.2 ms, non-improvers 1296.9 ± 37.3 ms, p = 0.01).

Patients with reduction in septal T1 over the study duration had evidence of reduced myocardial contractility at baseline, as their baseline GLS was reduced in magnitude compared to those whose T1 time did not fall (Improvers GLS −16.27 ± 4.38, non-improvers, GLS −21.05 ± 5.94, p = 0.04). By follow up, there was no significant difference in GLS between the groups.

Although overall LVMI was no different at baseline or follow up in septal T1 time improvers versus non-improvers, the difference in LVMI over during the study was greater in those whose T1 time fell (difference in LVMI improvers: −12.8 ± 10.4 g/m^2^, non-improvers: −3.3 ± 5.7 g/m^2^, p = 0.009).

There were no significant differences in hs-tropT, NT-proBNP, BP, BMI, age, duration of RRT, URR, mean UF volumes, corrected calcium or haemoglobin between those whose septal T1 time fell and those whose septal T1 time did not. Phosphate was higher at baseline and follow up in those whose septal T1 time fell (T1 improvers: baseline phosphate 1.92 ± 0.52, non-improvers 1.35 ± 0.33, p = 0.01). There was no difference in incidence of diagnosis of congestive cardiac failure, hypertension, ischemic heart disease, previous myocardial infarction or diagnosis of diabetes between those whose septal T1 fell and those whose septal T1 did not.

## Discussion

This is the first study to investigate effects of 6-months of HD on incident HD patients utilizing multi-parametric CMR with native T1 mapping and feature tracking myocardial strain analysis. We observed trends suggesting that there is an association between HD and an improvement in myocardial biomechanics (identified by feature-tracking derived GLS), and regression of LVH. These results require validation in a larger cohort of patients.

The improvements in GLS and strain rate are encouraging. Previous studies have shown that GLS measured using speckle tracking echocardiography correlates with other markers of uremic cardiomyopathy, including fibrosis on histology^15^. That regression in LVMI and improvement in GLS and strain rate occurred together is suggests that the higher the LVMI, the less efficient left ventricular contraction and relaxation are and that as LVMI regresses, the ventricle becomes more compliant. As suggested by correlation of reduction in septal T1 relaxation times with reduction in LVMI, our study findings may indicate that as LVMI regresses, fibrosis also regresses, resulting in improved myocardial biomechanics leading to enhancement in GLS and strain rate. However, without histological confirmation, this is speculative.

Regression of LVMI in incident HD patients has been demonstrated previously. Regression in LVMI over time in patients new to HD was shown in an observational echocardiography study, where LVMI regressed in 48% of patients following one year of HD^16^. Another study considered echocardiographic changes in patients with end stage renal disease and heart failure^17^. Echocardiography was performed up to 3 months prior to HD initiation and again 8.6 ± 5.2 months after starting HD. There was a significant reduction in LVMI (mean reduction 24.3 ± 35.4 g/m^2^). The authors concluded this was due to improvement in volume status^17^. Although improved volume status is possibly a factor in the reduction in LVMI seen in our study, we saw no changes in EDV or ESV. Additionally, mean ultrafiltration volumes for 30 days prior to imaging did not change from baseline to follow up. Supporting the notion that change in LVMI in our study was not purely related to volume changes, there was no change in patient weight from baseline to follow up. However, we cannot exclude that overall improvement in volume status contributed to regression in LVMI and improvements in myocardial strain. Conversely, a continued rise in LVMI after dialysis initiation has been demonstrated using echocardiography, however it may be that timing of imaging, changing volume status or other co-morbidities in previous work contributed to this difference^18^.

Additionally, our data do not support the theory that LVMI regression and improvement in GLS were due to better BP control, as there was no difference in BP from baseline to follow up. Similarly, other makers of dialysis efficacy such as URR, calcium and phosphate were unchanged. That the changes in imaging abnormalities are independent of BP is not entirely surprising. A CMR study comparing patients with CKD and hypertensive patients, showed that LVMI was highest in the CKD group and that GLS was worse in the CKD group despite the hypertensive group having the highest BP^12^.

We did not observe reductions in either global or septal T1 relaxation times over the study duration, although there was an absolute difference in magnitude of T1 time of 5 ms. A larger sample size, or longer follow up duration may have led to a statistically significant change in T1 time as there were some indications in the study that T1 is interrelated to other markers of tissue abnormality. In fact, baseline septal T1 correlated with both GLS and LVMI. Change in septal T1 time also correlated with the difference in LVMI over a 6-month period. However, despite supporting evidence from histological studies^19–21^, without tissue correlation, we cannot be certain that changes in LVMI, myocardial strain and T1 times are reflective of an improvement in myocardial tissue abnormalities.

Although this was a relatively small single centre study, improvements seen in CMR derived cardiac parameters are of potentially important clinical significance. Participants whose T1 times improved had higher baseline T1 relaxation times and poorer baseline GLS. The possibility of regression to the mean should be considered. To attempt to address this, we investigated the effect of paired measures and the effect of regression to the mean by ANCOVA analysis- p 0.001 for LVMI, and p < 0.001 for GLS. Reassuringly, even after weighting for within subject change, the difference in measurements was still significant. It is too early to tell whether the patients whose cardiac parameters improved will subsequently have reduced cardiac morbidity and mortality. However, there is a reasonable body of evidence that LVH in HD is associated with an increased cardiac morbidity, cardiac mortality and all-cause mortality^2,3^. Echocardiography derived GLS also predicts mortality in patients with CKD^22,23^.

Limitations of our study include it being single centre, and small sample size. Patients were recruited on average with a 5 month HD duration. Our power calculation was based on data using gadolinium, to reproduce our work it would be possible to base sample sizes on T1 data in dialysis patients^9,10^. However, we used a multi-parametric CMR protocol and biochemical markers so our study population was well characterized. Future studies would include a multicentre approach with larger sample size, and potentially with histological validation of T1.

In summary, this study demonstrated that in incident HD patients, 6 months of HD was associated with improved LVMI and myocardial strain. Although no significant difference in T1 times from baseline to follow up was seen, change in septal T1 time associated with change in LVMI. We consider that these findings reflect improvement in tissue abnormalities known to be associated with renal failure. However, without tissue correlation we cannot be certain - future studies with findings correlated with cardiac histology are of interest.

## Materials and Methods

### Study Participants

Thirty-four HD participants consented to participate in the Cardiac Uraemic fibrosis Detection in DiaLysis patiEnts study (CUDDLE study ISRCTN99591655). The West of Scotland Research Ethics Service (13/WS/0301) approved the study. The study was carried out in accordance with the declaration of Helsinki. Participants were eligible if they had been receiving HD for <12 months. Exclusion criteria included atrial fibrillation as this impairs cardiac-gating of images, or CMR contraindications^24^. All patients provided written, informed consent and received usual thrice weekly dialysis care for the study duration. Participants who underwent renal transplantation during follow up were withdrawn from the study.

### CMR Imaging Technique

All participants underwent CMR on a post-dialysis day on a 3 T scanner (MAGNETOM Verio, or Prisma, Siemens, Erlangen, Germany) at baseline and after 6-months of HD. Patients who underwent dialysis on a Monday/Wednesday/Friday schedule were imaged on a Tuesday or Thursday, Tuesday/Thursday/Saturday patients were imaged on Wednesdays or Fridays. A double radio-frequency array coil (anterior and posterior) was used. The imaging protocol included cine magnetic resonance with steady-state free precession and T1 mapping sequences^25^. A cine short-axis stack of the LV was performed. Basal, mid and apical T1 maps were acquired using motion-corrected, optimized, modified Look-Locker inversion recovery sequences without contrast. Typical T1 acquisition parameters were: slice thickness 6.0 mm, voxel size: 1.9 × 1.9 × 6.0 mm, field of view 340 mm × 272 mm, flip angle 35 degrees, minimum T1 180 ms, inversion-time increment 80 ms, repetition time 267.84 ms, bandwidth 1085 Hertz/pixel.

### Image Analysis

LV indices were analysed by a single blinded observer as previously described^9^. For T1 maps, LV contours were drawn onto colour-enhanced spatially co-registered maps. The anterior right ventricular insertion point was used as reference and T1 maps were segmented corresponding with the American Heart Association (AHA) 16-segment model^26^. Segmental AHA regions were delineated by user-defined border delineation on Siemens Argus software. T1 times were measured in each segment. Care was taken to ensure adequate margins from tissue interfaces such as between the blood pool and myocardium. After removal of any segments affected by artefact, global T1 time was calculated as the mean of remaining segments. Septal T1 time was calculated by averaging remaining AHA anteroseptal, inferoseptal, and septal segments (i.e. segments 2, 3, 8, 9, 14). To determine GLS, strain rate and end-diastolic strain rate feature-tracking software (TomTec, Diogenes Image Arena, Munich, Germany) was used. On horizontal long-axis cine acquisition, the end-diastolic frame was identified for each image and endocardial borders were delineated. The delineated contour was then automatically propagated throughout the cardiac cycle and GLS, strain rate and early diastolic strain rate calculated^27^.

### Biomarkers

Pre-dialysis blood samples at baseline and after 6-months follow up were frozen (−80 °C) and thawed for analysis of hs-tropT and NT-proBNP in a single batch using validated assays (e411, Roche Diagnostics, Burgess Hill, UK). All samples were analysed using manufacturers’ protocols and calibrations. Routine blood tests including haemoglobin, urea reduction ratios (URR), albumin, corrected calcium and phosphate were available from electronic patient records (EPR). EPR were used for medical and dialysis history including pre-and post-dialysis weights and ultrafiltration volumes 30 days prior to imaging.

### Statistics

Statistical analyses were performed using SPSS version 22 (Armonk, NY). Normality was tested using visual distribution checks and the Kolmogorov-Smirnov test. Paired t tests (for parametric data) and Wilcoxon-Signed Rank tests (for nonparametric data) were used to compare continuous indices between time points within HD patients. Independent t tests (for parametric data) and Mann-Whitney U tests (for nonparametric data) were used to compare between group differences. Correlations between continuous indices were assessed using Pearson’s and Spearman’s correlation coefficients for parametric and nonparametric data, respectively.

The power calculation was based on mass of fibrotic tissue detected with gadolinium as previously published^11^ as at commencement of this study design there were no data to inform distribution of T1 times in HD patients.

Data from patients studied with gadolinium showed that distribution of fibrosis, when present was normally distributed with standard deviation 7 g. If the true difference in mean difference in volume of fibrotic tissue detected at CMR in matched pairs is 4.5 g, we determined 27 patients were needed (paired t-test at 0 and 6 months) to detect this difference with power of 90% and type I error probability of 0.05. Allowing for a 20% dropout due to transplant, death and co-morbid disease, we aimed to recruit 35 patients.
